# Evaluating Cognitive Action Control Using Eye-Movement Analysis: An Oculomotor Adaptation of the Simon Task

**DOI:** 10.3389/fnhum.2016.00084

**Published:** 2016-03-02

**Authors:** Joan Duprez, Jean-François Houvenaghel, Florian Naudet, Thibaut Dondaine, Manon Auffret, Gabriel Robert, Dominique Drapier, Soizic Argaud, Marc Vérin, Paul Sauleau

**Affiliations:** ^1^“Behavior and Basal Ganglia” Research Unit (EA 4712), University of Rennes 1Rennes, France; ^2^Neurology Department, Rennes University HospitalRennes, France; ^3^Adult Psychiatry Department, Rennes University HospitalRennes, France; ^4^Clinical Investigation Center (INSERM 0203), Department of Pharmacology, Rennes University HospitalRennes, France; ^5^Neuroscience of Emotion and Affective Dynamics Lab, Swiss Center for Affective SciencesGeneva, Switzerland; ^6^Neurophysiology Department, Rennes University HospitalRennes, France

**Keywords:** cognitive control, conflict task, activation-suppression, distributional analysis, eye-movement

## Abstract

Cognitive action control has been extensively studied using conflict tasks such as the Simon task. In most recent studies, this process has been investigated in the light of the dual route hypothesis and more specifically of the activation-suppression model using distributional analyses. Some authors have suggested that cognitive action control assessment is not specific to response modes. In this study we adapted the Simon task, using oculomotor responses instead of manual responses, in order to evaluate whether the resolution of conflict induced by a two-dimensional stimulus yielded similar results to what is usually reported in tasks with manual responses. Results obtained from 43 young healthy participants revealed the typical congruence effect, with longer reaction times (RT) and lesser accuracy in the incongruent condition. Conditional accuracy functions (CAF) also revealed a higher proportion of fast errors in the incongruent condition and delta plots confirmed that conflict resolution was easier, as the time taken to respond increased. These results are very similar to what has been reported in the literature. Furthermore, our observations are in line with the assumptions of the activation-suppression model, in which automatic activation in conflict situations is captured in the fastest responses and selective inhibition of cognitive action control needs time to build up. Altogether, our results suggest that conflict resolution has core mechanisms whatever the response mode, manual or oculomotor. Using oculomotor responses in such tasks could be of interest when investigating cognitive action control in patients with severe motor disorders.

## Introduction

In a situation of conflicting response tendencies, we must be able to override activation of the inappropriate response and select the correct one. This process, often named *cognitive action control*, has been extensively studied via conflict tasks (Ridderinkhof et al., 2011). Several different conflict tasks have been used for this purpose, including the Eriksen flanker task, the Stroop task and the Simon task (Stroop, 1935; Simon, 1969; Eriksen and Eriksen, 1974; Wöstmann et al., 2013).

The use of two-dimensional stimuli is a common feature of some of these tasks such as the Stroop task or the Simon task. These stimuli contain both a relevant information, giving instructions on how to achieve the desired action, and an irrelevant information that might induce inappropriate responses in conflicting situations. In the Simon task, for instance, participants are presented with stimuli in the form of two colors that are displayed on a screen either on the left or the right side. Heeding the relevant-color information and ignoring the irrelevant-location information, participants have to choose a response by pressing one of two buttons as fast and as accurately as possible. In the congruent condition, both the relevant-color and the irrelevant-location information activate the same response. Conversely, in the incongruent situation, color and location induce opposite responses. These incongruent situations increase reaction times (RT) and decrease response accuracy, whereas congruent situations facilitate response. In the Stroop task the major difference is that both stimulus dimensions overlap (the color of the word’s ink and the meaning of the word are identical or not) whereas in the Simon task the stimulus irrelevant dimension (location) overlaps with the location of the response key. Nevertheless, it seems that both paradigms induce conflict in response selection (for a review, see Lu and Proctor, 1995).

In most studies, the difference between the congruent and the incongruent situations is known as the congruence effect (van den Wildenberg et al., 2010). This congruence effect is commonly explained by the dual-process hypothesis. It states that stimulus processing follows two parallel routes converging towards a given response activation: a direct route that leads to a fast and fairly automatic activation, and an indirect and controlled route that is slower and selects the most appropriate program to engage (Kornblum et al., 1990; De Jong et al., 1994; Eimer, 1995). An important property of the direct route is that it is activated according to overlearned stimulus-response associations, whereas response activation selected by the controlled route depends on intentions. When both direct and controlled routes lead to the same activation (congruent situation), the program first selected by the direct route is carried out rapidly. However, when the response selected by automatic activation mismatches the one selected by the controlled route (incongruent situation), the former has to be suppressed to allow correct response selection.

Ridderinkhof (2002) took this theory further and integrated cognitive action control into a dynamic model: the activation-suppression model. According to this model, an inappropriate behavioral response is actively suppressed by selective inhibition mechanisms. In case of conflicting response tendencies, an incorrect activation has to be canceled in favor of the correct response. However, suppressing and overcoming this incorrect response activation takes time. The activation-suppression model makes two important assumptions. When the response is too fast in incongruent situations, there is a higher probability of impulsive actions captured by the irrelevant dimension, and thus, a higher probability of error. The other phenomenon predicted by the model is that the cost of the congruence effect in terms of RT decreases when the time taken to respond is greater since effective inhibition builds up gradually.

The assumptions of this model can be tested using a distributional analysis. Recent studies have used conditional accuracy functions (CAF) for both congruent and incongruent trials, and delta plots which enable the representation of the level of accuracy and the congruence effect, respectively, as a function of RT (Ridderinkhof, 2002; Wylie et al., 2009a,b, 2010). More precisely, CAF are used to assess automatic response capture, whereas delta plots give insights into the effectiveness of the selective inhibition process. In the Simon task, these analyses typically show that rapid responses are more error-prone in the incongruent condition and that the congruence effect is less pronounced in slower responses as predicted by the activation-suppression model (van den Wildenberg et al., 2010). An alternative account to explain the decreasing congruence effect in the Simon task is that this decrease might result from a passive decay of the automatic activation (Hommel, 1993). However, strong arguments from electrophysiological studies support the view of an active selective inhibition mechanism (Hasbroucq et al., 2001; Burle et al., 2002). Eventually, the activation-suppression model and distributional analysis of conflict task results have been of great help in investigating cognitive control in both healthy and pathological populations in the recent years (Forstmann et al., 2008b; Wylie et al., 2009a,b, 2010, 2012; Fluchère et al., 2015). For instance, Wylie et al. (2009b) studied the effects of Parkinson’s disease on cognitive action control using a Simon task which was analyzed with CAF and delta plots according to the activation-suppression model.

Although most conflict tasks rely on manual responses (button presses; Ridderinkhof, 2002; Egner et al., 2007; Forstmann et al., 2008b), some studies have focused on the saccadic Stroop effect (Hodgson et al., 2009; Hermens and Walker, 2012). For instance, Hermens and Walker (2012) presented colored words at the center of the screen and participants had to make a saccade towards a peripheral colored patch. Incongruent situations arose when the meaning of the word indicated a different location than its color. They found the same congruence effect on RT and errors than in manual tasks. These results are in line with the study of Eimer and Schlaghecken (2001) using a priming paradigm who suggested that the dual route and the activation-suppression model were not specific to response mode and could be investigated using oculomotor responses. Further, a study from Sullivan and Edelman (2009) using a Simon task with both manual and saccadic responses showed the typical congruence effect in both modes. Moreover, Wijnen and Ridderinkhof (2007) reported similar mechanisms and dynamics for response inhibition in manual and oculomotor conflict tasks. In a variation of the oculomotor capture task, participants were asked to respond to a target in one hemifield while ignoring a salient distracting stimulus that could appear in the other hemifield with either a saccade or a button press. The authors showed that it was possible to study the congruence effect and the selective inhibition mechanism using oculomotor responses. Using the same task, the authors also showed that it was possible to assess automatic activation using the oculomotor mode (Wijnen and Ridderinkhof, 2000). Using CAF analysis of the eye movements, they confirmed that most errors were present in the fastest responses (first bin of the distribution). These data suggests a common top-down inhibition process in conflict resolution, whatever be the response mode. However, the analysis of conflict resolution induced by a two-dimensional stimulus such as in the Simon Task has not yet been performed, using both the oculomotor mode and distributional analyses. With this in mind, we chose to develop an adaptation of the Simon task, as this task has been extensively used and is known to yield sensitive results regarding cognitive action control (van den Wildenberg et al., 2010). We presented young healthy participants with a two-dimensional stimulus, one of which was relevant (color) and the other irrelevant (location). Participants had to effect a saccade toward the target side and to resolve conflict when the two dimensions required opposite responses. We interpreted our results on the basis of the activation-suppression model using distributional analysis.

## Materials and Methods

### Participants

The participants were 43 healthy volunteers (mean age ± *SD* = 23.7 ± 3.5 years; 27 women). Most of them were students from the University of Rennes (France), with a mean education level of 15.4 ± 1.4 years. Interviews were carried out prior to the task to ensure that the participants had no history of psychiatric or neurological disorders and no recent history of drug use. All participants reported normal or corrected-to-normal vision and provided informed consent in accordance with the Declaration of Helsinki. The experiment was conducted in accordance with the local Ethics Committee of Rennes University Hospital.

### Stimuli and Apparatus

**Figure 1:**
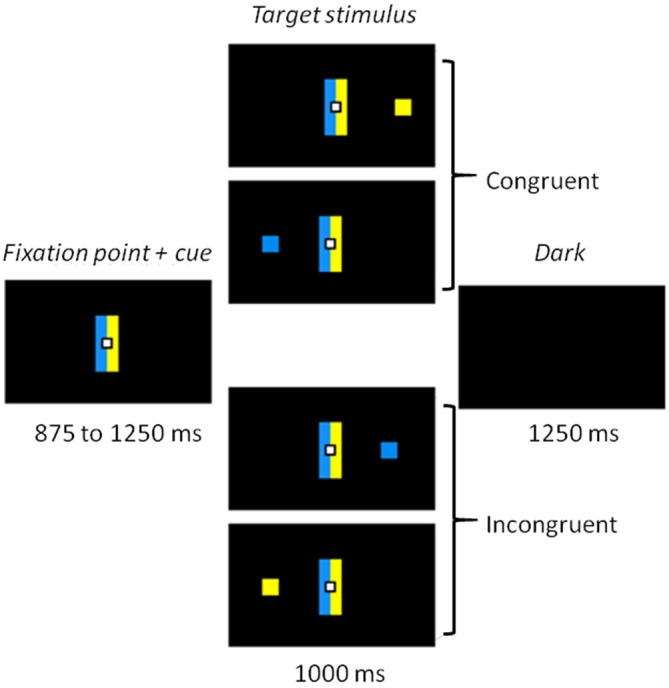
**Experimental task.** Participants had to make a left or right eye movement according to the color of the target stimulus while ignoring its location. When the side indicated by the color matched the location of the target, the trial was congruent. When color and location did not match, the trial was incongruent and a conflict arose.

Participants were sitting 60 cm away from of a 22-inch screen (screen frequency: 60 Hz). The whole task was conducted in the dark, to ensure the quality of the eye movement recordings, with dim light interruptions during the inter-block intervals. The task was designed using MeyeParadigm^®^ Software (Version 1.18, e(ye)BRAIN^1^). The stimuli were displayed on a black ground. A central white square (0.6 cm in height and width) was used as a fixation point, flanked by two contiguous rectangles (5 cm in height and 1 cm in width), one blue, one yellow, representing the cues (Figure 1). The color side of these cues was pseudo-randomly reversed across participants. The target of each trial was a blue or a yellow square (0.6 cm in height and width) presented at a 12° visual angle either on the left or on the right. The targets were displayed pseudo-randomly, with an equal number of 2 (color) × 2 (location) combinations. The task was divided into five 3.5 min blocks, each containing 60 trials, making a total of 300 trials. The blocks were separated by short breaks to prevent tiredness. Each trial started with the presentation of the central fixation point and the contiguous rectangles (cues) for 875–1250 ms (pseudorandom 125 ms steps). The target was then displayed for 1000 ms, either on the left or the right side of the screen. The trial ended with a black screen, which was displayed for 1250 ms before the next trial started. A practice run of 16 trials was performed before the experimental phase, in order to familiarize participants with the task.

### Task and Procedure

Participants were asked to make a left or right eye movement according to the color of the target (relevant information), ignoring its location (irrelevant information). They were instructed to perform the eye movement as fast and as accurately as possible. When the blue cue was on the left (for half of the participants), they were to effect a saccade to the left when the stimulus was blue and a saccade to the right when it was yellow (Figure 1). The instructions and the sides of the cues were reversed for the other half of the participants. Color-response mapping was balanced across participants, with half of them performing the task with blue indicating a left response and the other half performing the task with yellow indicating a left response. The cue rectangles on either side of the central fixation point remained on the screen when the stimulus was displayed, in order to keep the instructions available and avoid a heavy working memory load. The presence of this cue is similar to the color coding of the response keys in the Hedge and Marsh version of the Simon task that has been recently used with distributional analyses (Hedge and Marsh, 1975; Wylie et al., 2010, 2012). One could argue, as Simon et al. (1981) did, that in this case, conflict might arise from correspondence of the display (blue stimulus on the opposite side of the blue cue for example). However, such discussion does not seem of major concern since several studies suggested that conflict in the Simon task occurred whenever the response is spatially coded (for a review, see Lu and Proctor, 1995).

In our design, the automatic over-learned response consisted in eye movement toward the *location* of the target, whereas the controlled response was to direct the eyes toward the location indicated by the *color* of the target. Thus, the location and the color of the target induced two conditions: a congruent one, when the stimulus was located on the side corresponding to the saccade direction dictated by its color (a blue square displayed on the left side in our example) and an incongruent one when it appeared on the opposite side (a yellow square on the left).

### Eye Movement Recording

Eye movements were recorded using an EyeBrain T2^®^ head-mounted eyetracker (e(ye)BRAIN^®^^2^) at a sampling rate of 300 Hz and an angular resolution of 0.5°. Horizontal saccades were analyzed off line with MeyeAnalysis^®^ Software (e(ye)BRAIN^®^^2^). This software automatically detected the onset and offset of the saccade using a detection algorithm adapted from Nyström and Holmqvist (2010). We could thus automatically extract RT (i.e., saccade latency) and saccade direction for each trial.

### Data Analysis

We considered the very first eye movement after stimulus presentation as the participants’ response, whether it was toward or away from the target. We analyzed the results using a distributional analysis based on the activation-suppression model (Ridderinkhof, 2002). When a participant performed a saccade according to the color of the target (blue or yellow), and not its location (left or right side), it was counted as a correct response. Both the direction and the latency of eye movements were studied. We excluded saccades with an amplitude below 2° (to discard any micro-movements around the central fixation point), a latency below 100 ms (to discard any anticipated saccades; for a review, see Leigh and Zee, 1999) or above 1000 ms (corresponding to the target duration), and outlier latencies more than three standard deviations from the mean RT. With these parameters, 0.9% of the whole dataset was removed from the analyses.

To assess automatic response capture elicited by the irrelevant dimension, we used CAF providing accuracy levels according to RT (Ridderinkhof, 2002). To this end we plotted accuracy against RT distribution for congruent and incongruent trials. For each participant, RT were rank-ordered and split into seven bins (septiles) containing an equal number of trials, and the mean accuracy was plotted for each bin. To visualize the dynamics of selective suppression, we then created delta plots to show the interference effect (difference between congruent RT and incongruent RT in correct trials) as a function of RT. RT were split into seven bins (septiles), as we did for the CAF, and mean delta values were calculated and plotted against the RT distribution for each bin.

### Statistical Analysis

Data management and statistical analysis were performed using R^©^ Software (Version 3.1.0) with the nlme (José Pinheiro, 2014; S version) and lme4 (Bates et al., 2014) packages.

RT were compared between congruence conditions using a linear mixed model, considering a fixed effect of congruence and a random participant effect. Since accuracy is a binary parameter, it was compared using a nonlinear mixed model (with the same fixed and random effects). These models allowed us to work on the whole dataset and avoid the loss of power that occurs with averaging data, while taking inter-individual variability into account. The CAF analysis was performed according to the same nonlinear mixed model, to which we added a “bin” fixed effect, corresponding to the RT distribution septiles. This resulted in a 2 (congruence) × 7 (bins) analysis. Delta plots were analyzed using a linear mixed model on delta values with a fixed effect “bin” and a random participant effect. *Post hoc* Tukey tests were used for further analyses when significant effects were found. The adjusted *p*-values were obtained using the Tukey glht function from the multcomp package which uses individual *z*-test (Hothorn et al., 2008).

## Results

### RT and Accuracy

**Figure 2:**
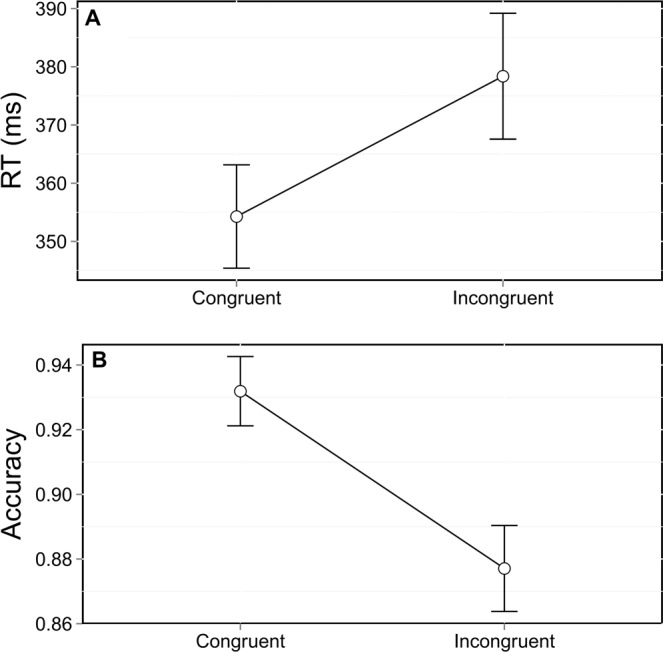
**Mean reaction times (RT; A) and accuracy (B) as a function of congruence.** Error bars represent the standard error of the mean.

As in the Simon task, and as commonly observed in other interference tasks, RT were strongly influenced by congruence, *F*_(1,11491)_ = 308.67, *p* < 0.0001. Participants were 25 ms slower in the incongruent trials (Figure 2A). Accuracy was also affected by congruence, *F*_(1,12753)_ = 114.09, *p* < 0.0001, with a 5% difference in accuracy between congruent and incongruent trials (Figure 2B).

### Distributional Analysis: Conditional Accuracy Functions

#### Conditional Accuracy Functions

**Figure 3:**
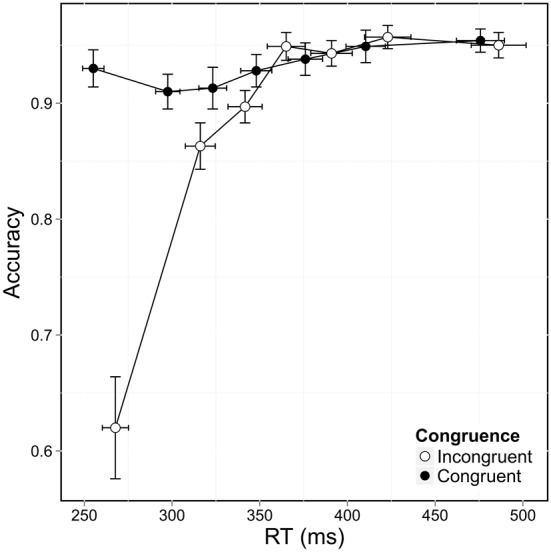
**Conditional accuracy functions (CAF) plotted for congruence as a function of RT distribution.** Error bars represent the standard error of the mean.

Accuracy differed according to congruence and according to bin, as shown when accuracy was plotted against RT distribution and congruence in CAF (Figure 3). Accuracy decreased markedly in the incongruent situation, *F*_(1,12741)_ = 125.144, *p* < 0.0001, and was also significantly lower for the fast responses, as shown by a significant bin effect, *F*_(6,12741)_ = 110.160, *p* < 0.0001. These two effects interacted significantly, *F*_(6,12741)_ = 97.749, *p* < 0.0001, and *post hoc* analysis showed that errors were mostly associated with the fastest RT (first two bins) in the incongruent condition. Accuracy was considerably lower in situations of conflict for the first bin (*z* = 16.2, *p* < 0.01), with an accuracy rate of 62% in the incongruent condition vs. 93% in the congruent condition. This difference persisted when congruence was compared for the second bin, but to a lesser extent (*z* = 3.4, *p* = 0.03), with accuracy rates of 86% (incongruent) vs. 91% (congruent). This effect disappeared when we focused on the other five bins (all *p*s > 0.05). This confirmed that most errors were made when participants responded very quickly in conflict situations. Accuracy for congruent trials remained above 90% across the RT distribution, meaning that participants were equally accurate, whether they responded quickly or slowly.

#### Delta Plots

**Figure 4:**
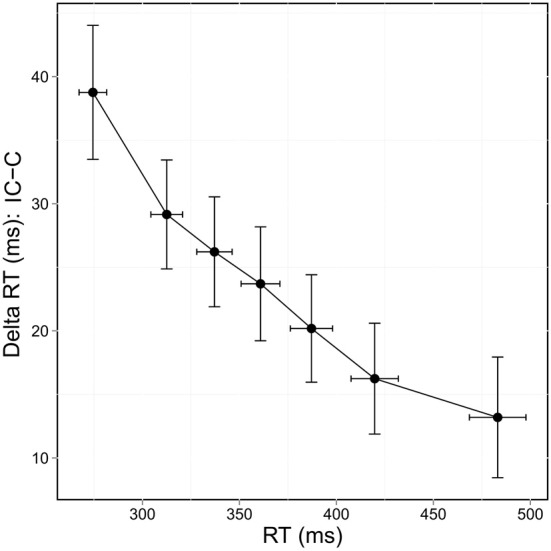
**Delta plots showing delta RT of correct responses (incongruent [IC] RT—congruent [C] RT) according to RT distribution**.

Delta values between congruent and incongruent RT in correct trials reflect the time needed to resolve conflict and delta plots reveal the evolution of these values across the bins of the RT distribution. The delta value between congruent and incongruent RT decreased as the time taken to respond increased (Figure 4). This was confirmed by the significant bin effect, *F*_(6,252)_ = 16.122, *p* < 0.0001.

## Discussion

The purpose of this study was to analyze the resolution of conflict induced by a two-dimensional stimulus, using an oculomotor adaptation of the Simon task test and distributional analysis. Our aim was to confirm that using the oculomotor mode yielded results consistent with the assumptions of the activation-suppression model (Ridderinkhof, 2002).

In our modified Simon task, participants were presented with a target stimulus featuring two dimensions: color and location. Young healthy participants were required to make a leftward or rightward eye movement based solely on the relevant color information of the target, ignoring its irrelevant location information. When the irrelevant and relevant information did not indicate the same response the trial was incongruent. These incongruent trials enabled us to assess the ability to suppress the response activated by the irrelevant dimension of the stimulus in favor of the one activated by the relevant dimension. Both the RT and the direction of the first eye movement after the stimulus presentation were used as the response.

Our results showed that when facing incongruent trials, participants needed more time to make a correct response and were more prone to making mistakes than in congruent trials. This congruence effect on RT and accuracy has repeatedly been reported in the literature across a range of conflict tasks using push-buttons (Stroop, 1935; Simon, 1969; Wallace, 1971; Eriksen and Eriksen, 1974; Hedge and Marsh, 1975; Egner et al., 2007; Forstmann et al., 2008b; Wöstmann et al., 2013). Furthermore, the size of the congruence effect on RT in our study was of 25 ms and matched results from previous studies that showed an effect of generally 20–30 ms (for a review, see Lu and Proctor, 1995). The congruence effect on errors seemed slightly higher in our study (5%) than in literature where it is often less marked (Burle et al., 2002; Ridderinkhof, 2002; Forstmann et al., 2008a,b). This could be explained by the use of oculomotor responses since it has been shown that saccadic responses in a Simon task yielded more errors than manual responses (Sullivan and Edelman, 2009). The results of our task are therefore in line with the dual route hypothesis, stating that response activation follows two parallel routes (Kornblum et al., 1990). The position of our stimulus activated the automatic route while its color activated the controlled route. When the two routes led to different responses there was a larger number of errors and correct responses were delayed.

Distributional analyses gave insights into the evolution of both the accuracy and the congruence effect according to the time taken to respond. We used these analyses to assess response capture and selective inhibition, respectively, as is usually done in the activation-suppression model. Our results on CAF showed that accuracy was strongly influenced by both congruence and RT bins. Compared to congruent trials, incongruent trials were more error-prone and most errors were found in the fastest responses as seen for the first bin of the CAF. When participants took more time to respond in the incongruent situation, accuracy was higher and reached the same level as with congruent trials by the end of the RT distribution. These results are consistent with studies on cognitive action control, using manual conflict tasks and distributional analyses (van den Wildenberg et al., 2010). Delta plots showed that the difference between congruent and incongruent RT decreased when the time taken to respond increased. In other words, the additional cost in terms of RT needed to give a correct response was lower when responses were slower. This result has been reported repeatedly in the literature for manual response modes in conflict tasks (De Jong et al., 1994; Burle et al., 2002; Wylie et al., 2009a,b).

All of our distributional analysis results are in line with the predictions of the activation-suppression model (Ridderinkhof, 2002). When conflict arises, an automatic activation of the incorrect response elicited by irrelevant information must be overruled in order to select the proper response. The model states that selective inhibition of the incorrect activation takes time to build up, with longer time being associated with stronger inhibition. When response is fast, the selective inhibition mechanism has not enough time to be effective and the automatic response is “captured” (van den Wildenberg et al., 2010). This was evidenced by the numbers of fast errors seen on the first bin in incongruent trials of our CAF. Moreover, on the delta plots we observed that the congruence effect decreased as the time taken to respond increased, indicating that the more time the participants took to respond, the less the irrelevant dimension of the target stimulus affected their response. This is in line with the assumption of the model that the strongest selective inhibition is associated with the longest RT.

However, it is important to note that the decreasing pattern of the congruence effect that we found seems restricted to some specific versions of the Simon task and is not observable in all conflict tasks (for a review, see Proctor et al., 2011). For instance, the arrow version of the Flanker task usually gives similar results compared to the Simon task regarding mean RT and accuracy but shows a different pattern of delta plot, with values that rather increase when RT increases (Wylie et al., 2009a). This increasing pattern has also been observed in the Stroop task (Pratte et al., 2010). Regarding the Simon task, the decreasing congruence effect only appeared in versions with right and left visual stimuli. Auditory versions or Simon tasks with vertical stimuli resulted in increasing congruence effects (Wascher et al., 2001; Wiegand and Wascher, 2005). Regarding these information, the activation-suppression model is well appropriate for explaining decreasing congruence effects but seems challenged when accounting for increasing patterns observed in some tasks. Thus, the explanation for the changes in the congruence effect across RT distribution is still a matter of debate and further research is needed.

Our adaption of the Simon task using eye movements reproduced the results of motor conflict tasks such as the Simon task or the Eriksen flanker task, and thus supports the assumptions of the activation-suppression model. The results of this study support the assumption that there is at least a partial common functioning of cognitive action control in both manual and oculomotor response modes (Eimer and Schlaghecken, 2001). This view has also been supported by research using other tasks such as the Stop-signal task which measures the inhibition of the ongoing response. Indeed, some studies proposed both a manual and an oculomotor version of the Stop-signal task (Logan and Irwin, 2000; Boucher et al., 2007). In these studies, the participants had to make hand or eye movements that had to be inhibited on some trials. The authors showed that despite some quantitative differences (stop-signal RT were shorter with eye-movements), the mechanisms involved in stopping the ongoing response were highly similar. This shared process makes sense, since manual and oculomotor control rely on common circuits involving structures such as the basal ganglia and frontal areas of the cortex (Hikosaka et al., 2000; McDowell et al., 2008; Ridderinkhof et al., 2011). However, a direct comparison of our tasks results with those derived from a classic Simon task within the same group of participants would be of great help in clarifying the similarities and differences between the two types of response and thus in clarifying the mechanisms of cognitive action control.

## Conclusion

Conflict tasks have often been used to assess cognitive action control in both healthy and pathological populations (Gastaldo et al., 2002; Ridderinkhof, 2002; Forstmann et al., 2008b; Wylie et al., 2009a,b, 2013). In recent studies, the results are now analyzed in the light of the activation-suppression model using distributional analyses. Furthermore, conflict tasks and distributional analyses are also used in studies involving functional neuroimaging, where brain activity is correlated with CAF results and delta plots (Forstmann et al., 2008a,b). Since our adaption of the Simon task yielded similar results to those commonly reported in the literature, we suggest that this task could be useful in the investigation of cognitive action control in both healthy and pathological populations. Indeed, our results are fully consistent with the activation-suppression model, which is increasingly used in studies focusing on cognitive action control and its temporal dynamics. Furthermore, our oculomotor adaptation of the Simon task could be of interest in assessing patients with severe motor disorders, and difficulties in performing on a classic manual version.

## Author Contributions

We state that the authors made the following contributions: JD: study conception, data acquisition, data analysis, data interpretation, drafting, final approval. J-FH: study conception, data interpretation, draft revision, final approval. FN: study conception, data interpretation, draft revision, final approval. TD: study conception, data interpretation, draft revision, final approval. MA: data acquisition, draft revision, final approval. GR: data interpretation, draft revision, final approval. DD: data interpretation, draft revision, final approval. MV: data interpretation, draft revision, final approval. SA: data acquisition, draft revision, final approval. PS: study conception, data interpretation, draft revision, final approval.

## Conflict of Interest Statement

The authors declare that the research was conducted in the absence of any commercial or financial relationships that could be construed as a potential conflict of interest.

## Footnotes

^1^www.eye-brain.com

^2^www.eye-brain.com

## Abbreviations

RT: Reaction times
CAF: Conditional accuracy functions.

## References

[B1] Bates D. Maechler M. Bolker B. Walker S. Christensen R. H. B. Singmann H. (2014). lme4: linear mixed-effects models using Eigen and S4 (Version 1.1–7). Available online at: http://cran.r-project.org/web/packages/lme4/index.html

[B2] Boucher L. Stuphorn V. Logan G. D. Schall J. D. Palmeri T. J. (2007). Stopping eye and hand movements: are the process independent? Percept. Psychophys. 69, 785–801. 10.3758/bf03193779 17929700

[B3] Burle B. Possamaï C.-A. Vidal F. Bonnet M. Hasbroucq T. (2002). Executive control in the Simon effect: an electromyographic and distributional analysis. Psychol. Res. 66, 324–336. 10.1007/s00426-002-0105-6 12466929

[B4] De Jong R. Liang C.-C. Lauber E. (1994). Conditional and unconditional automaticity: a dual-process model of effects of spatial stimulus-response correspondence. J. Exp. Psychol. Hum. Percept. Perform. 20, 731–750. 10.1037/0096-1523.20.4.731 8083631

[B5] Egner T. Delano M. Hirsch J. (2007). Separate conflict-specific cognitive control mechanisms in the human brain. Neuroimage 35, 940–948. 10.1016/j.neuroimage.2006.11.061 17276088

[B6] Eimer M. (1995). Stimulus-response compatibility and automatic response activation: evidence from psychophysiological studies. J. Exp. Psychol. Hum. Percept. Perform. 21, 837–854. 10.1037//0096-1523.21.4.837 7643051

[B7] Eimer M. Schlaghecken F. (2001). Response facilitation and inhibition in manual, vocal and oculomotor performance: evidence for a modality-unspecific mechanism. J. Mot. Behav. 33, 16–26. 10.1080/00222890109601899 11265054

[B8] Eriksen B. A. Eriksen C. W. (1974). Effects of noise letters upon the identification of a target letter in a nonsearch task. Percept. Psychophys. 16, 143–149. 10.3758/10.3758/BF03203267

[B9] Fluchère F. Deveaux M. Burle B. Vidal F. van den Wildenberg W. P. Witjas T.. (2015). Dopa therapy and action impulsivity: subthreshold error activation and suppression in Parkinson’s disease. Psychopharmacology 232, 1735–1746. 10.1007/s00213-014-3805-x 25510855

[B10] Forstmann B. U. Jahfari S. Scholte H. S. Wolfensteller U. van den Wildenberg W. P. Ridderinkhof K. R. (2008a). Function and structure of the right inferior frontal cortex predict individual differences in response inhibition: a model-based approach. J. Neurosci. 28, 9790–9796. 10.1523/JNEUROSCI.1465-08.2008 18815263 PMC6671204

[B11] Forstmann B. van den Wildenberg W. Ridderinkhof K. (2008b). Neural mechanisms, temporal dynamics and individual differences in interference control. J. Cogn. Neurosci. 20, 1854–1865. 10.1162/jocn.2008.20122 18370596

[B12] Gastaldo S. Umiltà C. Bianchin G. Prior M. (2002). The simon effect in schizophrenic patients with negative symptoms. Cortex 38, 149–159. 10.1016/s0010-9452(08)70647-6 12056686

[B13] Hasbroucq T. Burle B. Akamatsu M. Vidal F. Possamaï C.-A. (2001). An electromyographic investigation of the effect of stimulus-response mapping on choice reaction time. Psychophysiology 38, 157–162. 10.1111/1469-8986.3810157 11321617

[B14] Hedge A. Marsh N. W. A. (1975). The effect of irrelevant spatial correspondences on two-choice response-time. Acta Psychol. 39, 427–439. 10.1016/0001-6918(75)90041-4 1199779

[B15] Hermens F. Walker R. (2012). The site of interference in the saccadic stroop effect. Vision Res. 73, 10–22. 10.1016/j.visres.2012.09.017 23026013

[B16] Hikosaka O. Takikawa Y. Kawagoe R. (2000). Role of the basal ganglia in the control of purposive saccadic eye movements. Physiol. Rev. 80, 953–978. 10893428 10.1152/physrev.2000.80.3.953

[B17] Hodgson T. L. Parris B. A. Gregory N. J. Jarvis T. (2009). The saccadic stroop effect: evidence for involuntary programming of eye movements by linguistic cues. Vision Res. 49, 569–574. 10.1016/j.visres.2009.01.001 19183561 PMC2724027

[B18] Hommel B. (1993). The relationship between stimulus processing and response selection in the Simon task: evidence for a temporal overlap. Psychol. Res. 55, 280–290. 10.1007/bf00419688

[B19] Hothorn T. Bretz F. Westfall P. (2008). Simultaneous inference in general parametric models. Biom. J. 50, 346–363. 10.1002/bimj.200810425 18481363

[B20] José Pinheiro (S version), Douglas Bates (up to 2007), Saikat DebRoy (up to 2002), Deepayan Sarkar (up to 2005), EISPACK authors (src/rs.f), Siem Heisterkamp, Bert Van Willigen, and R-core (2014). nlme: linear and nonlinear mixed effects models (Version 3.1–124). Available online at: http://cran.r-project.org/web/packages/nlme/index.html

[B21] Kornblum S. Hasbroucq T. Osman A. (1990). Dimensional overlap: cognitive basis for stimulus-response compatibility-a model and taxonomy. Psychol. Rev. 97, 253–270. 10.1037//0033-295x.97.2.253 2186425

[B22] Leigh R. J. Zee D. S. (1999). The Neurology of Eye Movements. (Vol. 90). New York, NY: Oxford University Press Available online at: http://www.oftalmo.com/seo/archivos/maquetas/5/34C7D31D-ED18-1669-3B1D-00001D182185/articulo.html

[B23] Logan G. D. Irwin D. E. (2000). Don’t look! don’t touch! Inhibitory control of eye and hand movements. Psychon. Bull. Rev. 7, 107–112. 10.3758/bf03210728 10780023

[B24] Lu C.-H. Proctor R. W. (1995). The influence of irrelevant location information on performance: a review of the Simon and spatial stroop effects. Psychon. Bull. Rev. 2, 174–207. 10.3758/BF03210959 24203654

[B25] McDowell J. E. Dyckman K. A. Austin B. P. Clementz B. A. (2008). Neurophysiology and neuroanatomy of reflexive and volitional saccades: evidence from studies of humans. Brain Cogn. 68, 255–270. 10.1016/j.bandc.2008.08.016 18835656 PMC2614688

[B26] Nyström M. Holmqvist K. (2010). An adaptive algorithm for fixation, saccade and glissade detection in eyetracking data. Behav. Res. Methods 42, 188–204. 10.3758/BRM.42.1.188 20160299

[B27] Pratte M. S. Rouder J. M. Morey R. D. Feng C. (2010). Exploring the differences in distributional properties between stroop and Simon effects using delta plots. Atten. Percept. Psychophys. 72, 2013–2025. 10.3758/APP.72.7.2013 20952797

[B28] Proctor R. W. Miles J. D. Baroni G. (2011). Reaction time distribution analysis of spatial correspondence effects. Psychon. Bull. Rev. 18, 242–266. 10.3758/s13423-011-0053-5 21327376

[B29] Ridderinkhof K. R. Forstmann B. U. Wylie S. A. Burle B. van den Wildenberg W. P. M. (2011). Neurocognitive mechanisms of action control: resisting the call of the Sirens. Wiley Interdiscip. Rev. Cogn. Sci. 2, 174–192. 10.1002/wcs.99 26302009

[B30] Ridderinkhof K. R. (2002). Micro-and macro-adjustments of task set: activation and suppression in conflict tasks. Psychol. Res. 66, 312–323. 10.1007/s00426-002-0104-7 12466928

[B31] Simon J. R. (1969). Reactions toward the source of stimulation. J. Exp. Psychol. 81, 174–176. 10.1037/h0027448 5812172

[B32] Simon J. R. Sly P. E. Vilapakkam S. (1981). Effect of compatibility of SR mapping on reactions toward the stimulus source. Acta Psychol. 47, 63–81. 10.1016/0001-6918(81)90039-1 7211438

[B33] Stroop J. R. (1935). Studies of interference in serial verbal reactions. J. Exp. Psychol. 18, 643–662. 10.1037/h0054651

[B34] Sullivan K. Edelman J. (2009). An oculomotor Simon effect. J. Vis. 9:380 10.1167/9.8.380

[B35] van den Wildenberg W. P. M. Wylie S. A. Forstmann B. U. Burle B. Hasbroucq T. Ridderinkhof K. R. (2010). To head or to heed? Beyond the surface of selective action inhibition: a review. Front. Hum. Neurosci. 4:222. 10.3389/fnhum.2010.00222 21179583 PMC3004391

[B36] Wallace R. J. (1971). SR compatibility and the idea of a response code. J. Exp. Psychol. 88, 354–360. 10.1037/h0030892 5090926

[B37] Wascher E. Schatz U. Kuder T. Verleger R. (2001). Validity and boundary conditions of automatic response activation in the Simon task. J. Exp. Psychol. Hum. Percept. Perform. 27, 731–751. 10.1037/0096-1523.27.3.731 11424658

[B38] Wiegand K. Wascher E. (2005). Dynamic aspects of stimulus-response correspondence: evidence for two mechanisms involved in the Simon effect. J. Exp. Psychol. Hum. Percept. Perform. 31, 453–464. 10.1037/0096-1523.31.3.453 15982125

[B39] Wijnen J. G. Ridderinkhof K. R. (2000). Age differences in oculomotor capture by highly salient objects revealed in conditional capture functions. Role Sel. Inhib. Adapt. Oculomot. Control 77, 87–109.

[B40] Wijnen J. G. Ridderinkhof K. R. (2007). Response inhibition in motor and oculomotor conflict tasks: different mechanisms, different dynamics? Brain Cogn. 63, 260–270. 10.1016/j.bandc.2006.09.003 17069944

[B41] Wöstmann N. M. Aichert D. S. Costa A. Rubia K. Möller H.-J. Ettinger U. (2013). Reliability and plasticity of response inhibition and interference control. Brain Cogn. 81, 82–94. 10.1016/j.bandc.2012.09.010 23174432

[B42] Wylie S. A. Claassen D. O. Huizenga H. M. Schewel K. D. Ridderinkhof K. R. Bashore T. R.. (2012). Dopamine agonists and the suppression of impulsive motor actions in Parkinson disease. J. Cogn. Neurosci. 24, 1709–1724. 10.1162/jocn_a_00241 22571461 PMC3657467

[B43] Wylie S. A. Claassen D. O. Kanoff K. E. Ridderinkhof K. R. van den Wildenberg W. P. (2013). Impaired inhibition of prepotent motor actions in patients with Tourette syndrome. J. Psychiatry Neurosci. 38, 349–356. 10.1503/jpn.120138 23820185 PMC3756119

[B44] Wylie S. A. Ridderinkhof K. R. Elias W. J. Frysinger R. C. Bashore T. R. Downs K. E.. (2010). Subthalamic nucleus stimulation influences expression and suppression of impulsive behaviour in Parkinson’s disease. Brain 133, 3611–3624. 10.1093/brain/awq239 20861152 PMC2995881

[B45] Wylie S. A. van den Wildenberg W. P. M. Ridderinkhof K. R. Bashore T. R. Powell V. D. Manning C. A.. (2009a). The effect of Parkinson’s disease on interference control during action selection. Neuropsychologia 47, 145–157. 10.1016/j.neuropsychologia.2008.08.001 18761363 PMC4524676

[B46] Wylie S. A. van den Wildenberg W. P. M. Ridderinkhof K. R. Bashore T. R. Powell V. D. Manning C. A.. (2009b). The effect of speed-accuracy strategy on response interference control in Parkinson’s disease. Neuropsychologia 47, 1844–1853. 10.1016/j.neuropsychologia.2009.02.025 19428416 PMC4524649

